# Allele frequencies for 40 autosomal SNP loci typed for US population samples using electrospray ionization mass spectrometry

**DOI:** 10.3325/cmj.2013.54.225

**Published:** 2013-06

**Authors:** Kevin M. Kiesler, Peter M. Vallone

**Affiliations:** Biomolecular Measurement Division, National Institute of Standards and Technology, Gaithersburg, MD, USA

## Abstract

**Aim:**

To type a set of 194 US African American, Caucasian, and Hispanic samples (self-declared ancestry) for 40 autosomal single nucleotide polymorphism (SNP) markers intended for human identification purposes.

**Methods:**

Genotyping was performed on an automated commercial electrospray ionization time-of-flight mass spectrometer, the PLEX-ID. The 40 SNP markers were amplified in eight unique 5plex PCRs, desalted, and resolved based on amplicon mass. For each of the three US sample groups statistical analyses were performed on the resulting genotypes.

**Results:**

The assay was found to be robust and capable of genotyping the 40 SNP markers consuming approximately 4 nanograms of template per sample. The combined random match probabilities for the 40 SNP assay ranged from 10^−16^ to 10^−21^.

**Conclusion:**

The multiplex PLEX-ID SNP-40 assay is the first fully automated genotyping method capable of typing a panel of 40 forensically relevant autosomal SNP markers on a mass spectrometry platform. The data produced provided the first allele frequencies estimates for these 40 SNPs in a National Institute of Standards and Technology US population sample set. No population bias was detected although one locus deviated from its expected level of heterozygosity.

The forensic community has addressed the application of autosomal single nucleotide polymorphism (SNP) markers for human identification (1-4). SNPs may be of utility when working with highly degraded DNA because they can be assayed with very small polymerase chain reaction (PCR) amplicons. Over the past 10 years, various SNP assays and candidate marker panels have been described (5-10). One set of interest is a panel of 40 autosomal SNP markers intended as a universal individual identification panel. These markers were selected for high heterozygosity and low F_st_ values in studies of 40 populations to complement CODIS STR loci (8). Initially these markers were screened and typed for world populations by singleplex TaqMan-based assays. More recently, there have been attempts to develop multiplex assays for typing the 40 SNP panel (11). One of these is the PLEX-ID SNP-40 comprised of 8 unique 5plex PCRs developed by Abbott Molecular. The PLEX-ID instrument platform is a commercial electrospray ionization mass spectrometer capable of automated analysis of short PCR amplicons (less than 140 bp) generated by proprietary assays (see SNP-40, mtDNA 2.0). The instrument desalts each PCR reaction through the use of magnetic bead chemistry and injects the desalted PCR reaction into the mass spectrometer. The peaks are separated and resolved based on time-of-flight analysis. With the emerging development of ultra-high throughput sequencing applied to forensics it will be more commonplace to utilize these “core” SNP maker sets. Here we report the assay performance and allele frequencies for a subset of our National Institute of Standards and Technology (NIST) US population samples.

## Methods

For this study, samples (n = 194) were selected from three population groups representative of major population segments in the United States (African Americans = 74, Caucasians = 75, and Hispanics = 45). 

Whole blood with anonymous identifiers and self-described ancestry was obtained from commercial blood banks (Interstate Blood Bank, Memphis, TN and Millennium Biotech, Fort Lauderdale, FL, USA). Blood samples were subjected to bulk DNA extraction using a modified salt-out procedure as described previously (12). DNA concentrations in extracts were determined using Quantifiler Human DNA Quantification kit (Life Technologies, Carlsbad, CA, USA) on an Applied Biosystems model 7500 (Life Technologies) real-time PCR instrument. Quantification values were then used to normalize all DNA extracts to a final concentration of 0.1 ng/µL for PCR amplification. All samples were previously examined with 15 autosomal short tandem repeats and the amelogenin sex-typing marker using the AmpFlSTR Identifiler kit (Applied Biosystems, Foster City, CA, USA) to verify that each sample was unique (13). 

### SNP typing

The 40 SNP markers typed by the PLEX-ID SNP-40 assay were previously selected and characterized on multiple world populations (8). The following data were obtained for the 40 SNP markers typed in the assay: dbSNP reference SNP (rs) number, nucleotide position (according to the Human February 2009 (GRCh37/hg19) assembly), chromosomal band, and physical distance from adjacent markers located on the same chromosome (Table 1).

**Table 1:** Information for the 40 autosomal single nucleotide polymorphism (SNP) loci examined in this study sorted by chromosome position. Chromosome positions were determined using the UCSC Genome Browser using Human Feb. 2009 (GRCh37/hg19) assembly

Marker	Chromosome	Nucleotide position	Chromosomal band	Physical distance from adjacent marker (nt)
rs7520386	1	14,155,402	1p36.21	
rs560681	1	160,786,670	1q23.3	146,631,268
rs1109037	2	10,085,722	2p25.1	
rs12997453	2	182,413,259	2q31.3	172,327,537
rs6444724	3	193,207,380	3q29	
rs279844	4	46,329,655	4p12	
rs13134862	4	76,425,896	4q21.1	30,096,241
rs1554472	4	157,489,906	4q32.1	81,064,010
rs6811238	4	169,663,615	4q32.3	12,173,709
rs13182883	5	136,633,338	5q31.2	
rs7704770	5	159,487,953	5q33.3	22,854,615
rs315791	5	169,735,920	5q35.1	10,247,967
rs338882	5	178,690,725	5q35.3	8,954,805
rs13218440	6	12,059,954	6p24.1	
rs1336071	6	94,537,255	6q16.1	82,477,301
rs1478829	6	120,560,694	6q22.31	26,023,439
rs1358856	6	123,894,978	6q22.31	3,334,284
rs2503107	6	127,463,376	6q22.33	3,568,398
rs447818	6	145,868,996	6q24.3	18,405,620
rs2272998	6	148,761,456	6q24.3	2,892,460
rs214955	6	152,697,706	6q25.2	3,936,250
rs1019029	7	13,894,276	7p21.2	
rs321198	7	137,029,838	7q33	123,135,562
rs10092491	8	28,411,072	8p21.1	
rs3780962	10	17,193,346	10p13	
rs1410059	10	97,172,595	10q24.1	79,979,249
rs740598	10	118,506,899	10q25.3	21,334,304
rs6591147	11	105,912,984	11q22.3	
rs10488710	11	115,207,176	11q23.3	9,294,192
rs1058083	13	100,038,233	13q32.3	
rs1821380	15	39,313,402	15q14	
rs7205345	16	7,520,254	16p13.3	
rs9951171	18	9,749,879	18p11.22	
rs7229946	18	22,739,001	18q11.2	12,989,122
rs985492	18	29,311,034	18q12.1	6,572,033
rs445251	20	15,124,933	20p12.1	
rs2567608	20	23,017,082	20p11.21	7,892,149
rs1523537	20	51,296,162	20q13.2	28,279,080
rs2073383	22	23,802,171	22q11.23	
rs987640	22	33,559,508	22q12.3	9,757,337

PCR amplification was performed as recommended by the manufacturer by adding a total of 0.5 ng in a 5 µL-volume of template DNA to each of eight wells in a column of a pre-fabricated SNP-40 assay plate (Abbott Molecular, Des Plaines, IL, USA). In total eight unique 5plex reactions were run per sample requiring approximately 4 ng of DNA template per sample. Template DNA was added to each well by using a pipette tip to pierce the foil seal covering the well to which sample was added. On each 96-well plate, ten unique templates were run in parallel with a no-template control and a positive amplification control, 9947a DNA, (Promega Corp., Madison, WI, USA) at 0.1 ng/µL. After template addition, the PCR plate was re-sealed using PCR Foil seals (Abbott Molecular) on an ALPS 50V Heat Sealer (ThermoFisher Scientific, Waltham, MA, USA) by compressing the foil seal and PCR plate for 4 seconds at 180°C. The prepared 96-well plate was then briefly centrifuged and placed in a Mastercycler ProS thermal cycler (Eppendorf AG, Hamburg, Germany) for thermal cycling with the following program: initial denaturation at 96°C for 10 minutes; 40 cycles of denaturation at 96°C for 20 seconds, annealing at 58.5°C for 2 minutes, and extension at 72°C for 10 seconds; followed by a final extension step at 72°C for 4 minutes and a 4°C hold.

Following PCR amplification, the 96-well plate was briefly centrifuged and placed in the input stacker of the PLEX-ID instrument for automated desalting and mass determination as per manufacturer’s recommended procedure.

PCR products were purified by the PLEX-ID instrument using a proprietary magnetic bead chemistry to remove salts, enzymes, unincorporated nucleotides, and any other PCR components that might interfere with collection of mass spectra. Purified PCR product was eluted in a buffer containing two peptide standards with masses of 727.4 Da and 1347.7 Da, which act as calibrants to facilitate data processing. The electrospray ionization source operates in negative mode at approximately -4000 V (depending on the individual instrument’s tuning parameters, which are not user configurable) and 300°C. PCR products were sprayed into the ionization source at a flow rate of 280 µL per hour with dry compressed air used as a countercurrent to aid in analyte desolvation. The time-of-flight analyzer collects 5000 scans per second, for a period of approximately 28 seconds. Masses were resolved based on differences in time elapsed to traverse the flight tube due to mass-to-charge ratio (m/z). Resultant mass spectra were processed by proprietary software (Ibis Track version 2.7), which performs several steps to produce a background-subtracted, deconvolved representation of the mass spectral data as if only the singly charged mass peak were detected, with mass (Daltons) on the x-axis and signal strength (arbitrary units) on the y-axis. Successfully detected masses were stored in a table that resides in the Ibis Track database. The resulting mass spectra were inspected visually in IbisTrack software and any masses not correctly assigned by the software were manually added or deleted.

Following review, genotypes for each sample group were exported from Ibis Track to Microsoft Excel 2010 for formatting and further analysis with Power Marker, version 3.25 and Arlequin software, version 3.5.1.2 (14,15). Allele frequencies, expected and observed heterozygosity values, and *P* values (Fisher exact test for Hardy-Weinberg equilibrium) for each marker were calculated for the three US sample groups. The combined random match probabilities (RMP) for each sample were calculated using Excel 2010.

## Results

**Figure 1:**
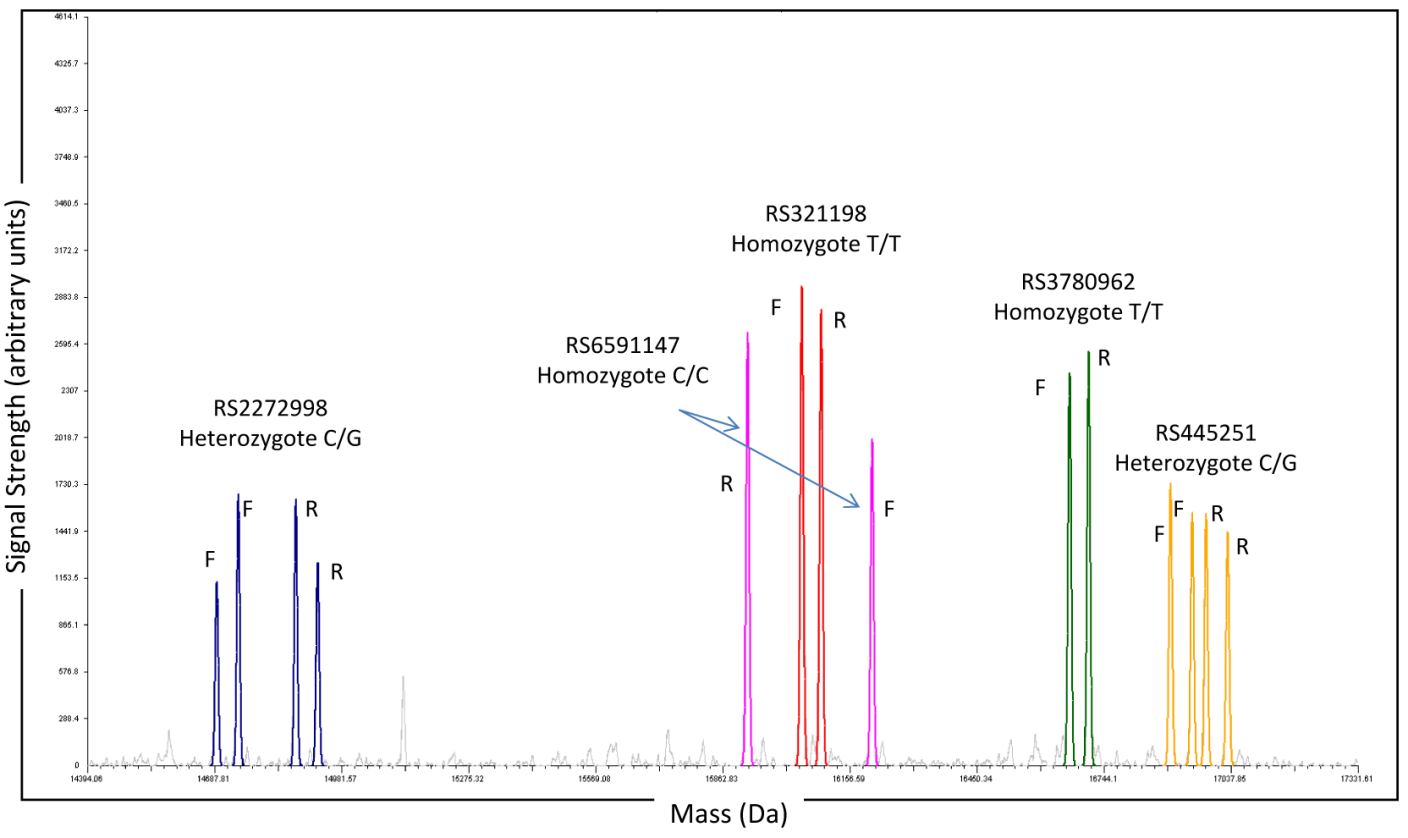
An example mass spectrum is shown. This specific example contains 3 homozygous (rs6591147, rs321198, rs3780962) and 2 heterozygous (rs2272998 and rs445251) markers. The forward and reverse strands for each polymerase chain reaction amplicon are highlighted. Note the mass interleaving of single nucleotide polymorphisms (SNP) rs6591147 and rs321198.

Figure 1 illustrates an example mass spectrum obtained from this study. Each spectrum contains the products of a 5plex PCR reaction. Four signal peaks are typically observed for heterozygous loci, two forward and two reverse strands of DNA (see markers rs2272998 and rs445251 in Figure 1). Only two signals peaks are observed for homozygous loci (see markers rs6591147, rs321198, and rs3780962). Of the 194 samples examined in this study, incomplete or partial genotypes were observed for 21 loci (21/7760 = 0.27%). Ten of the failures were due to data not transferring to the PLEX-ID server due to a communication error. The remaining incomplete genotypes coincided with amplification reactions that exhibited poor signal over the entire 5plex. This may have been due to inefficient PCR or desalting in those specific amplification reactions. There was no evidence of a single locus dropping out due to underlying SNPs that would affect PCR primer binding.

The genotype data for the 194 samples was evaluated for the following parameters: allele frequencies, observed heterozygosity, expected heterozygosity, and *P* value (Table 2). The combined RMP for each sample was calculated based on the determined allele frequencies for the corresponding sample group (Table 3).

**Table 2:** Allele frequencies observed for the 3 US sample groups listed by single nucleotide polymorphism (SNP) locus. Each SNP is identified with the corresponding dbSNP rs number. The format for each allele is listed in the line above the frequency. Example rs10092491 [C/T] where A = C and B = T. The *P* values less than 5% for the markers rs1019029, rs1358856, rs6811238, rs1523537, rs447818, and rs13182883 are bolded

	Afr Amer	Cauc	Hisp	Afr Amer	Cauc	Hisp	Afr Amer	Cauc	Hisp	Afr Amer	Cauc	Hisp	Afr Amer	Cauc	Hisp
	rs10092491 [C/T]			rs1019029 [C/T]			rs10488710 [C/G]			rs1058083 [A/G]			rs1109037 [A/G]		
**He**	0.503	0.494	0.485	0.500	0.503	0.485	0.496	0.477	0.490	0.501	0.474	0.468	0.503	0.503	0.506
**Hob**	0.575	0.467	0.489	0.479	0.733	0.578	0.554	0.427	0.467	0.527	0.413	0.545	0.507	0.547	0.378
**fA**	0.479	0.567	0.600	0.459	0.487	0.400	0.561	0.613	0.589	0.466	0.380	0.364	0.514	0.513	0.500
**fB**	0.521	0.433	0.400	0.541	0.513	0.600	0.439	0.387	0.411	0.534	0.620	0.636	0.486	0.487	0.500
***P*-value**	0.1546	0.6288	1.0000	0.6425	**0.0001**	0.2150	0.2272	0.4631	0.7510	0.6435	0.3191	0.3400	1.0000	0.4956	0.1320
	**rs12997453 [A/G]**			**rs13134862 [A/G]**			**rs13182883 [A/G]**			**rs13218440 [A/G]**			**rs1336071 [A/G]**		
**He**	0.441	0.494	0.463	0.492	0.464	0.475	0.503	0.488	0.502	0.462	0.417	0.497	0.458	0.494	0.475
**Hob**	0.486	0.600	0.444	0.419	0.560	0.400	0.432	0.427	0.318	0.438	0.373	0.556	0.397	0.440	0.356
**fA**	0.324	0.433	0.356	0.426	0.360	0.378	0.486	0.413	0.455	0.356	0.293	0.433	0.349	0.433	0.378
**fB**	0.676	0.567	0.644	0.574	0.640	0.622	0.514	0.587	0.545	0.644	0.707	0.567	0.651	0.567	0.622
***P*-value**	0.4391	0.1040	1.0000	0.1492	0.0840	0.3470	0.1515	0.3523	**0.0230**	0.6126	0.4118	0.5650	0.2884	0.3453	0.1140
	**rs1358856 [A/C]**			**rs1410059 [C/T]**			**rs1478829 [A/T]**			**rs1523537 [C/T]**			**rs1554472 [C/T]**		
**He**	0.503	0.503	0.499	0.480	0.502	0.497	0.500	0.486	0.481	0.477	0.468	0.493	0.502	0.502	0.503
**Hob**	0.568	0.640	0.533	0.405	0.467	0.467	0.486	0.467	0.467	0.365	0.467	0.400	0.459	0.547	0.533
**fA**	0.514	0.507	0.444	0.608	0.473	0.567	0.541	0.593	0.611	0.385	0.367	0.422	0.527	0.473	0.467
**fB**	0.486	0.493	0.556	0.392	0.527	0.433	0.459	0.407	0.389	0.615	0.633	0.578	0.473	0.527	0.533
***P*-value**	0.3532	**0.0222**	0.7660	0.2234	0.4888	0.7510	0.8134	0.8144	1.0000	**0.0261**	0.7980	0.2000	0.4775	0.3641	0.7560
	**rs1821380 [C/G]**			**rs2073383 [C/T]**			**rs214955 [A/G]**			**rs2272998 [C/G]**			**rs2503107 [A/C]**		
**He**	0.463	0.483	0.502	0.498	0.490	0.502	0.496	0.503	0.493	0.492	0.468	0.490	0.479	0.490	0.493
**Hob**	0.554	0.480	0.511	0.595	0.467	0.556	0.608	0.520	0.568	0.446	0.493	0.467	0.562	0.573	0.489
**fA**	0.358	0.400	0.456	0.554	0.580	0.544	0.561	0.487	0.420	0.426	0.367	0.411	0.610	0.580	0.578
**fB**	0.642	0.600	0.544	0.446	0.420	0.456	0.439	0.513	0.580	0.574	0.633	0.589	0.390	0.420	0.422
***P*-value**	0.0804	1.0000	1.0000	0.1058	0.8075	0.5460	0.0614	0.8210	0.3590	0.3412	0.6295	0.7660	0.1675	0.0947	1.0000
	**rs2567608 [A/G]**			**rs279844 [A/T]**			**rs315791 [A/C]**			**rs321198 [C/T]**			**rs338882 [C/T]**		
**He**	0.503	0.503	0.497	0.501	0.498	0.506	0.496	0.503	0.481	0.488	0.496	0.505	0.500	0.503	0.502
**Hob**	0.548	0.560	0.422	0.554	0.493	0.600	0.466	0.520	0.556	0.500	0.466	0.378	0.507	0.560	0.422
**fA**	0.479	0.520	0.567	0.466	0.553	0.500	0.562	0.513	0.611	0.588	0.562	0.522	0.459	0.507	0.544
**fB**	0.521	0.480	0.433	0.534	0.447	0.500	0.438	0.487	0.389	0.412	0.438	0.478	0.541	0.493	0.456
***P*-value**	0.3494	0.3567	0.3770	0.4816	1.0000	0.2420	0.6339	0.8214	0.3430	0.8141	0.6342	0.1700	0.8112	0.2645	0.3640
	**rs3780962 [C/T]**			**rs445251 [C/G]**			**rs447818 [A/G]**			**rs560681 [A/G]**			**rs6444724 [C/T]**		
**He**	0.500	0.498	0.505	0.477	0.490	0.485	0.496	0.490	0.497	0.415	0.433	0.425	0.494	0.496	0.505
**Hob**	0.541	0.493	0.444	0.473	0.452	0.489	0.658	0.440	0.511	0.419	0.387	0.422	0.405	0.533	0.556
**fA**	0.541	0.553	0.511	0.385	0.418	0.400	0.438	0.420	0.433	0.709	0.687	0.700	0.568	0.440	0.522
**fB**	0.459	0.447	0.489	0.615	0.582	0.600	0.562	0.580	0.567	0.291	0.313	0.300	0.432	0.560	0.478
***P*-value**	0.6343	1.0000	0.5570	0.8014	0.4720	1.0000	**0.0047**	0.4735	1.0000	1.0000	0.4299	1.0000	0.1564	0.6400	0.5500
	**rs6591147 [C/T]**			**rs6811238 [G/T]**			**rs7205345 [C/G]**			**rs7229946 [A/G]**			**rs740598 [A/G]**		
**He**	0.503	0.464	0.490	0.485	0.494	0.505	0.500	0.500	0.485	0.500	0.499	0.497	0.487	0.452	0.481
**Hob**	0.527	0.480	0.422	0.486	0.360	0.444	0.514	0.520	0.622	0.479	0.480	0.511	0.466	0.493	0.511
**fA**	0.520	0.640	0.589	0.595	0.567	0.511	0.541	0.540	0.600	0.459	0.453	0.433	0.589	0.660	0.611
**fB**	0.480	0.360	0.411	0.405	0.433	0.489	0.459	0.460	0.400	0.541	0.547	0.567	0.411	0.340	0.389
***P*-value**	0.8144	0.7994	0.3610	0.8135	**0.0211**	0.5700	1.0000	0.6288	0.0700	0.6553	0.8186	1.0000	0.6048	0.3051	0.7620
	**rs7520386 [A/G]**			**rs7704770 [A/G]**			**rs985492 [C/T]**			**rs987640 [A/T]**			**rs9951171 [A/G]**		
**He**	0.503	0.500	0.493	0.480	0.490	0.499	0.503	0.496	0.497	0.503	0.488	0.449	0.490	0.503	0.502
**Hob**	0.466	0.573	0.578	0.486	0.440	0.477	0.541	0.480	0.511	0.473	0.507	0.578	0.459	0.560	0.600
**fA**	0.507	0.540	0.578	0.608	0.580	0.557	0.514	0.440	0.433	0.480	0.413	0.333	0.419	0.493	0.544
**fB**	0.493	0.460	0.422	0.392	0.420	0.443	0.486	0.560	0.567	0.520	0.587	0.667	0.581	0.507	0.456
**p-value**	0.6534	0.1666	0.3550	1.0000	0.4948	1.0000	0.5155	0.8242	1.0000	0.6496	0.8243	0.1150	0.6274	0.2435	0.2270

*Abbreviations: He – expected heterozygosity; Hob – observed heterozygosity; fA – frequency of allele A; fB – frequency of allele B; *P*-value – Fisher exact test for Hardy-Weinberg equilibrium; format of marker.

**Table 3:** Summary of random match probabilities calculated (RMP) for the three sample groups. The median values, minimum, and maximum observed RMPs for each sample group are listed.

Combined random match probability	African American	Caucasian	Hispanic
**Median**	3.37E-18	2.53E-18	2.29E-18
**Minimum**	2.15E-20	4.09E-21	2.86E-21
**Maximum**	2.39E-16	4.76E-16	7.35E-17

## Discussion

A total of 6 of the 120 tests (40 loci ×3 sample groups) for Hardy-Weinberg equilibrium indicated a deviation from the expected result. Three were observed in the Caucasian sample group (rs1019029, rs1358856, and rs6811238), 2 in the African American group (rs1523537 and rs447818), and 1 in the Hispanic group (rs13182883). It was shown that it can be expected to observe approximately 5%, or 6 out of 120, of the comparisons to deviate from Hardy-Weinberg equilibrium (16,17). Significant values at the 95% confidence level were those less than 0.05 (5%). The Bonferroni correction of probability for each population was 0.05/40 = 0.00125. Using this criterion only the SNP marker rs1019029 would still be considered significant.

Typically the minimum number of samples needed to provide a robust estimate for allele frequencies with loci containing 5 to 15 alleles is 100 to 150 samples for each population (18). Since in this study we measured bi-allelic markers that only had three possible genotypes (AA, BB, or AB), a smaller number of samples is deemed to be sufficient – provided that a minimum allele frequency of 5/2N is utilized (19). An examination of the data for each sample group did not find any frequency measurements (out of the 360 total) below the 5/2N threshold.

In the Caucasian sample group, the SNP marker rs1019029 exhibited a low *P* value (<0.0001) as well as a high observed heterozygosity of 0.733. The same marker gave an observed heterozygosity of 0.472 in the study by Pakstis et al over the 40 populations examined (8). An additional review of the mass spectral data did not reveal an obvious error with the genotyping assay. The high heterozygosity was not observed in the African American or Hispanic sample groups, suggesting that testing additional Caucasian samples and/or an alternate typing method would be needed to confirm this result.

The median combined RMP across the 3 US sample groups was approximately 2.73 × 10^−18^ with a minimum of 2.39 × 10^−16^ (African American sample) and maximum of 2.86 × 10^−21^ (Hispanic sample). Pakstis et al reported an average RMP across 40 populations of 10^−16^ with a range of 2.02 × 10^−17^ to 1.29 × 10^−13^ (8).

This is the first demonstration of a multiplex assay for typing this specific panel of 40 autosomal SNPs. We found the PLEX-ID instrument and SNP-40 assay to be a robust and automated method to type SNP markers. The time required to genotype 40 SNPs for 10 samples from start to finish (PCR to amplicon detection) was approximately 4.5 hours. The average time required to review a plate (10 samples plus positive and negative controls) in the IbisTrack software was approximately 15 minutes. The allele frequencies calculated for the US sample groups were found to be in agreement with published values, with the possible exception of rs1019029. The allele frequencies are the first derived from the NIST US populations sample set for this panel of 40 SNP markers intended as a universal panel for individual identification (8).

**Access to the data:** Genotyping results are available at: *http://www.cstl.nist.gov/biotech/strbase/NISTpop.htm*.
